# Supplementary material for: The Evolution and Characterization of the RNA Interference Pathways in Lophotrochozoa
Source: Genome Biol Evol. 2024 May 7;16(5):evae098. doi: 10.1093/gbe/evae098 (PMC11114477; doi:10.1093/gbe/evae098)
Supplement: evae098_Supplementary_Data [file evae098_supplementary_data.zip › Supplementary_materials.zip!/selected_miRNAs/NoGe_selected_miRNAs/mirdeep_pdf_files/NMRB01000823.1_Notospermus_geniculatus_strain_Ushimado_scaffold823,_whole_genome_shotgun_sequence_21770.pdf]

Provisional ID	:	NMRB01000823.1_Notospermus_geniculatus_strain_Ushimado_scaffold823,_whole_genome_shotgun_sequence_21770
Score total	:	2747.8
Score for star read(s)	:	3.9
Score for read counts	:	2738.9
Score for mfe	:	2
Score for randfold	:	0
Score for cons. seed	:	3
Total read count	:	5384
Mature read count	:	5273
Loop read count	:	0
Star read count	:	111

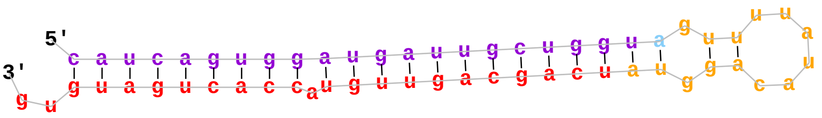

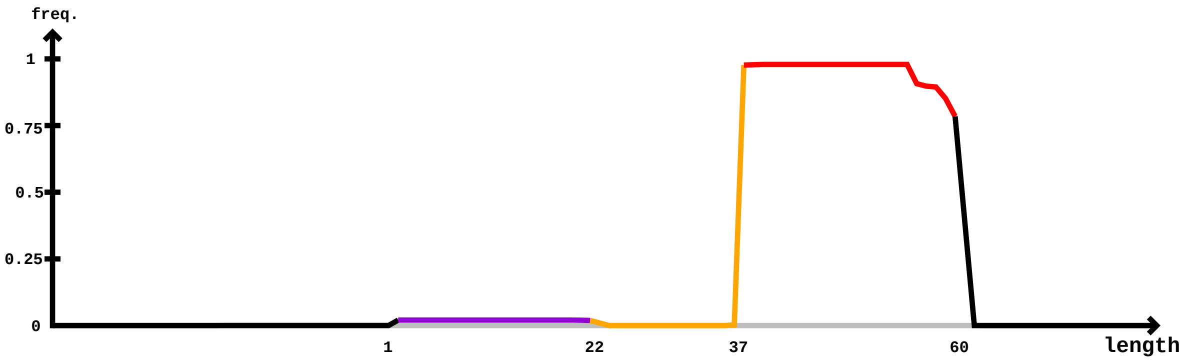


```
                                                   Star                              Mature
5'- caucagugcaagaauaaaauuuugucguagcccuccaucaguggaugauugcugguaguuuuauacagguaucagcaguuguaccacugaugugggacauugacacaggcaac   -3'   obs
    caucagugcaagaauaaaauuuugucguagcccuccaucaguggaugauugcugguaguuuuauacagguaucagcaguuguaccacugaugugggacauugacacaggcaac         exp
    ......(((..(..........(((((...((((.((((((((((((((((((((((.((......)).))))))))))))).))))))))).))))....))))))..)))..    reads    mm    sample
    ......................................................................Cucagcaguuguaccacugaugug....................    10       1     Fm2
    .......................................................................ucagcaguuguaccacuA.........................    1        1     Fm2
    .......................................................................ucagcaguuguaccacug.........................    7        0     Fm2
    .......................................................................ucagcaguuguaccacuga........................    2        0     Fm2
    .......................................................................ucagcaguCguaccacugaug......................    1        1     Fm2
    .......................................................................ucagcaguuguaccacugaug......................    33       0     Fm2
    .......................................................................ucagcaguuguaccacuCaugu.....................    1        1     Fm2
    .......................................................................ucagcaguuguaccacugaugu.....................    33       0     Fm2
    .......................................................................ucagcaguuguaccacugauguA....................    19       1     Fm2
    .......................................................................ucagcaguuguaccacuAaugug....................    6        1     Fm2
    .......................................................................ucagcaguuguaccacuCaugug....................    4        1     Fm2
    .......................................................................ucagcaguuguaccCcugaugug....................    1        1     Fm2
    .......................................................................uAagcaguuguaccacugaugug....................    1        1     Fm2
    .......................................................................ucagcGguuguaccacugaugug....................    2        1     Fm2
    .......................................................................ucagcaguugCaccacugaugug....................    1        1     Fm2
    .......................................................................ucagcaguuguaccacugauguU....................    17       1     Fm2
    .......................................................................ucagcaguuguacUacugaugug....................    2        1     Fm2
    .......................................................................ucagcaguuguaccacGgaugug....................    3        1     Fm2
    .......................................................................Ncagcaguuguaccacugaugug....................    1        1     Fm2
    .......................................................................ucagcaguuguaccacugaugug....................    1178     0     Fm2
    .......................................................................ucagcaguuguaccacAgaugug....................    3        1     Fm2
    .......................................................................ucaCcaguuguaccacugaugug....................    1        1     Fm2
    .......................................................................ucagcaguuguaccacCgaugug....................    6        1     Fm2
    .......................................................................ucagcaguugGaccacugaugug....................    3        1     Fm2
    .......................................................................ucagcaguuguaccacuUaugug....................    4        1     Fm2
    .......................................................................ucagcaguuguaccacuAaugugg...................    1        1     Fm2
    .......................................................................ucagcaguuguaccacugaugugg...................    19       0     Fm2
    .......................................................................ucagcaguuguaccacugaugugU...................    35       1     Fm2
    ........................................................................cagcaguuguaccacugaugug....................    4        0     Fm2
    .........................................................................agcaguuguaccacugaugug....................    2        0     Fm2

    ...................................caucaguggaugauugcugg...........................................................    4        0     Fm1
    ...................................caucaguggaugauugcuggu..........................................................    19       0     Fm1
```

```
                                  Star                                 Mature
caucagugcaagaauaaauuuugucguagcccuccaucaguggaugauugcugguaguuuuauacagguaucagcaguuguaccacugaugugggacauugacacaggcaac
..................................caucaguggaugauugcuggua.........................................................   12     0   Fm1
.....................................................................Cucagcaguuguaccacugaugug....................   1      1   Fm1
......................................................................ucagcaguuguaccacGg.........................   1      1   Fm1
......................................................................ucagcaguuguaccacug.........................   44     0   Fm1
......................................................................ucagcaguuguaccacuga........................   2      0   Fm1
......................................................................ucagcaguuguaccacugau.......................   7      0   Fm1
......................................................................ucagcaguuguaccacuCaug......................   1      1   Fm1
......................................................................ucagcaguuguaccacugaug......................   62     0   Fm1
......................................................................Ncagcaguuguaccacugaugu.....................   1      1   Fm1
......................................................................ucagcaguuguaccacugaugu.....................   112    0   Fm1
......................................................................ucagcaguuguaccacuAaugu.....................   1      1   Fm1
......................................................................ucagcaguuguGccacugaugug....................   1      1   Fm1
......................................................................ucagcaguuguaccacugaugGg....................   2      1   Fm1
......................................................................ucagcaguuguacAacugaugug....................   1      1   Fm1
......................................................................ucagcaguuguaccacugaugug....................   1039   0   Fm1
......................................................................ucGgcaguuguaccacugaugug....................   1      1   Fm1
......................................................................ucagcaguuguaccacugauguC....................   2      1   Fm1
......................................................................ucagcaguuguaccacuUaugug....................   5      1   Fm1
......................................................................ucagcaguuguaGcacugaugug....................   1      1   Fm1
......................................................................ucagcaguugAaccacugaugug....................   2      1   Fm1
......................................................................ucagcaguuguaccacuCaugug....................   6      1   Fm1
......................................................................ucagcaguuguaccacugauguU....................   27     1   Fm1
......................................................................ucagcaguuguaccacugauguA....................   34     1   Fm1
......................................................................ucagcaguuguaccacGgaugug....................   3      1   Fm1
......................................................................ucagcaguuguaccacuAaugug....................   7      1   Fm1
......................................................................ucagcaguugGaccacugaugug....................   3      1   Fm1
......................................................................ucagcaguuguaccacugCugug....................   1      1   Fm1
......................................................................ucaAcaguuguaccacugaugug....................   1      1   Fm1
......................................................................ucagcaguuguaccacCgaugug....................   1      1   Fm1
......................................................................ucagcaguuguaccacugaugugg...................   30     0   Fm1
......................................................................ucagcaguuguaccacugaugugU...................   96     1   Fm1
......................................................................ucagcaguuguaccacuUaugugg...................   1      1   Fm1
......................................................................ucagcaguuguaccacuCaugugg...................   1      1   Fm1
......................................................................ucagcaguuguaccacugauguggU..................   1      1   Fm1

................aauuuugucguagcccuc...............................................................................   1      0   Mm2
..................................caucaguggaugauugcugg...........................................................   1      0   Mm2
..................................caucaguggaugauugcuggu..........................................................   7      0   Mm2
......................................................................ucagcaguuguaccacug.........................   22     0   Mm2
......................................................................ucagcaguuguaccacuU.........................   1      1   Mm2
......................................................................ucagcaguuguaccacugaug......................   9      0   Mm2
......................................................................uUagcaguuguaccacugaugu.....................   1      1   Mm2
......................................................................ucagcaguuguaccacugaugu.....................   19     0   Mm2
......................................................................ucagcaguuguaccacuAaugu.....................   1      1   Mm2
......................................................................ucagcaguuguaccacuCaugug....................   1      1   Mm2
......................................................................ucagcaguuguaccUcugaugug....................   1      1   Mm2
......................................................................ucagcaguuguaccacugaugug....................   100    0   Mm2
......................................................................ucagcaguuguaUcacugaugug....................   1      1   Mm2
......................................................................ucagcaguuguaccacugauguU....................   4      1   Mm2
......................................................................ucagcaguuguaccacugaugugU...................   21     1   Mm2
......................................................................ucagcaguuguaccacugaugugg...................   18     0   Mm2
......................................................................ucagcaguuguaccacugaugugA...................   2      1   Mm2
......................................................................ucagcaguuUuaccacugaugugg...................   1      1   Mm2

..................................caucaguggaugaCugcuggua.........................................................   1      1   Mm1
..................................caucaguggaugauugcuggua.........................................................   14     0   Mm1
......................................................................ucagcaguuguaccacug.........................   56     0   Mm1
......................................................................ucagcaguuguaccacuC.........................   1      1   Mm1
......................................................................ucagcaguuguaccacuga........................   8      0   Mm1
......................................................................ucagcaguuguaccacugauU......................   10     1   Mm1
......................................................................ucagcaguuguaccacugaugu.....................   48     0   Mm1
......................................................................uAagcaguuguaccacugaugug....................   1      1   Mm1
......................................................................ucagcaguuguaccacuUaugug....................   2      1   Mm1
......................................................................ucagcaguuguaccacuAaugug....................   1      1   Mm1
......................................................................ucagcaguuguaccacugaugug....................   248    0   Mm1
......................................................................ucGgcaguuguaccacugaugug....................   1      1   Mm1
......................................................................ucagcaguuguaccacuCaugug....................   1      1   Mm1
......................................................................ucagcaguuUuaccacugaugug....................   1      1   Mm1
......................................................................ucagcaguuguaccacGgaugug....................   1      1   Mm1
......................................................................Ncagcaguuguaccacugaugug....................   1      1   Mm1
```

```
                                  Star                                Mature
caucagugcaagaauaaauuuugucguagcccuccaucaguggaugauugcugguaguuuuauacagguaucagcaguuguaccacugauguggggacauugacacaggcaac
......................................................................ucagcaguuguaccacugauguU....................    3    1    Mm1
......................................................................ucagcaguugAaccacugaugug....................    1    1    Mm1
......................................................................ucagcaguuguaccacuCaugugg...................    1    1    Mm1
......................................................................ucagcaguuguaccacugaugugg...................    54   0    Mm1
......................................................................ucagcaguuguaccacugaugugU...................    162  1    Mm1

..................................caucaguggaugauugcug............................................................    3    0    Fm3
..................................caucaguggaugauugcAggu..........................................................    1    1    Fm3
..................................caucaguggaugauugcuggu..........................................................    40   0    Fm3
..................................caucaguggaugauugcuggua.........................................................    3    0    Fm3
..................................caucaguggaugauugAuggua.........................................................    1    1    Fm3
..................................caucaguggaugauugcGggua.........................................................    1    1    Fm3
..................................caucaguggaugauugcAggua.........................................................    1    1    Fm3
.....................................................................Cucagcaguuguaccacuga........................    1    1    Fm3
......................................................................ucagcaguuguaccacuA.........................    1    1    Fm3
......................................................................ucagcaguuguaccacuU.........................    1    1    Fm3
......................................................................uUagcaguuguaccacug.........................    1    1    Fm3
......................................................................ucagcaguugCaccacug.........................    1    1    Fm3
......................................................................ucagcaguuguaccacug.........................    224  0    Fm3
......................................................................ucagcaguuguaccacuC.........................    1    1    Fm3
......................................................................ucagcaguuguaccacuga........................    33   0    Fm3
......................................................................ucagcaguuguaccacugau.......................    7    0    Fm3
......................................................................ucagcaguuguaUcacugaug......................    1    1    Fm3
......................................................................ucagcagCuguaccacugaug......................    1    1    Fm3
......................................................................ucagcaguugGaccacugaug......................    1    1    Fm3
......................................................................ucagcaguugCaccacugaug......................    1    1    Fm3
......................................................................ucagcaguuguaccacugaug......................    94   0    Fm3
......................................................................ucagcaguuguaccacuCaug......................    3    1    Fm3
......................................................................ucagcNguuguaccacugaugu.....................    1    1    Fm3
......................................................................ucagcaguuguaccacCgaugu.....................    2    1    Fm3
......................................................................ucagcaguuguaccacugaugu.....................    129  0    Fm3
......................................................................ucagcaguuguaccacuCaugu.....................    3    1    Fm3
......................................................................ucagcaguugAaccacugaugug....................    2    1    Fm3
......................................................................ucagcaguuguaccacGgaugug....................    5    1    Fm3
......................................................................ucagcagAuguaccacugaugug....................    1    1    Fm3
......................................................................ucagcaguuguaccacugaugug....................    667  0    Fm3
......................................................................ucagcaguuguaUcacugaugug....................    1    1    Fm3
......................................................................ucagcaguuguaccacAgaugug....................    1    1    Fm3
......................................................................ucagcaguuguacAacugaugug....................    1    1    Fm3
......................................................................ucagcaguuguaccacuAaugug....................    5    1    Fm3
......................................................................ucagcaguuguaccacuUaugug....................    1    1    Fm3
......................................................................ucagcaguuguaccacugauguU....................    14   1    Fm3
......................................................................ucagcaguuguaccaUugaugug....................    8    1    Fm3
......................................................................ucagcaguuguaccacCgaugug....................    2    1    Fm3
......................................................................ucagcaguCguaccacugaugug....................    1    1    Fm3
......................................................................ucagcaguuguaccacuCaugug....................    1    1    Fm3
......................................................................ucagcaguuguaccacugauguA....................    15   1    Fm3
......................................................................uAagcaguuguaccacugaugug....................    1    1    Fm3
......................................................................ucagcaguuguaccacugaugugg...................    37   0    Fm3
......................................................................ucagcaguuguaccacuCaugugg...................    1    1    Fm3
......................................................................ucagcaguuguaccacugaugugC...................    1    1    Fm3
......................................................................ucagcaguuguaccacugaugugA...................    5    1    Fm3
......................................................................ucagcaguuguaccacugaugugU...................    153  1    Fm3
.......................................................................cagcaguuguaccacugaug......................    1    0    Fm3
.......................................................................cagcaguuguaccacugaugug....................    2    0    Fm3
........................................................................agcaguuguaccacugaugug....................    1    0    Fm3

..................................caucaguggaugauugcuggu..........................................................    3    0    Mm3
......................................................................ucagcaguuguaccacAg.........................    1    1    Mm3
......................................................................ucagcaguuguaccacug.........................    25   0    Mm3
......................................................................ucagcaguuguaccacuga........................    5    0    Mm3
......................................................................ucagcaguuguaccacugau.......................    2    0    Mm3
......................................................................ucagcaguuguaccacugaug......................    12   0    Mm3
......................................................................ucagcaguuguaccacuAaug......................    1    1    Mm3
......................................................................ucagcaguuguaccacugaugu.....................    11   0    Mm3
......................................................................ucagcaguuguaccacugauguU....................    1    1    Mm3
......................................................................ucagcaguuguaccacuAaugug....................    1    1    Mm3
......................................................................ucagcaguuguaccacugaugug....................    45   0    Mm3
......................................................................ucagcaguuguaccacugauguA....................    4    1    Mm3
......................................................................ucagcaguuguaccacugaugugA...................    1    1    Mm3
```

Star **Mature**

```
caucagugcaagaauaaauuuugucguagcccuccaucaguggaugauugcugguaguuuuauacagguaucagcaguuguaccacugauguggggacauugacacaggcaac
......................................................................ucagcaguuguaccacugaugugU...................   18   1   Mm3
......................................................................ucagcaguuguaccacugaugugg...................   8    0   Mm3
........................................................................agcaguuguaccacugaugug....................   3    0   Mm3
```
